# TLR7 is expressed by support cells, but not sensory neurons, in ganglia

**DOI:** 10.1186/s12974-021-02269-x

**Published:** 2021-09-16

**Authors:** Becky J. Proskocil, Karol Wai, Katherine M. Lebold, Mason A. Norgard, Katherine A. Michaelis, Ubaldo De La Torre, Madeline Cook, Daniel L. Marks, Allison D. Fryer, David B. Jacoby, Matthew G. Drake

**Affiliations:** 1 Division of Pulmonary and Critical Care Medicine, Oregon Health & Science University, 3181 SW Sam Jackson Park Road, UHN67, Portland, OR 97239 USA; 2 Papé Family Pediatric Research Institute, Oregon Health & Science University, Portland, OR USA

**Keywords:** Dorsal root ganglia, Iba1 (ionized calcium-binding adaptor molecule 1), GFAP (glial fibrillary acidic protein), Influenza A, Sensory nerve, TLR7 (Toll-like receptor 7), Vagal ganglia

## Abstract

**Background:**

Toll-like receptor 7 (TLR7) is an innate immune receptor that detects viral single-stranded RNA and triggers the production of proinflammatory cytokines and type 1 interferons in immune cells. TLR7 agonists also modulate sensory nerve function by increasing neuronal excitability, although studies are conflicting whether sensory neurons specifically express TLR7. This uncertainty has confounded the development of a mechanistic understanding of TLR7 function in nervous tissues.

**Methods:**

TLR7 expression was tested using in situ hybridization with species-specific RNA probes in vagal and dorsal root sensory ganglia in wild-type and TLR7 knockout (KO) mice and in guinea pigs. Since TLR7 KO mice were generated by inserting an *Escherichia coli* lacZ gene in exon 3 of the mouse TLR7 gene, wild-type and TLR7 (KO) mouse vagal ganglia were also labeled for lacZ. In situ labeling was compared to immunohistochemistry using TLR7 antibody probes. The effects of influenza A infection on TLR7 expression in sensory ganglia and in the spleen were also assessed.

**Results:**

In situ probes detected TLR7 in the spleen and in small support cells adjacent to sensory neurons in the dorsal root and vagal ganglia in wild-type mice and guinea pigs, but not in TLR7 KO mice. TLR7 was co-expressed with the macrophage marker Iba1 and the satellite glial cell marker GFAP, but not with the neuronal marker PGP9.5, indicating that TLR7 is not expressed by sensory nerves in either vagal or dorsal root ganglia in mice or guinea pigs. In contrast, TLR7 antibodies labeled small- and medium-sized neurons in wild-type and TLR7 KO mice in a TLR7-independent manner. Influenza A infection caused significant weight loss and upregulation of TLR7 in the spleens, but not in vagal ganglia, in mice.

**Conclusion:**

TLR7 is expressed by macrophages and satellite glial cells, but not neurons in sensory ganglia suggesting TLR7’s neuromodulatory effects are mediated indirectly via activation of neuronally-associated support cells, not through activation of neurons directly. Our data also suggest TLR7’s primary role in neuronal tissues is not related to antiviral immunity.

**Supplementary Information:**

The online version contains supplementary material available at 10.1186/s12974-021-02269-x.

## Introduction

Toll-like receptor 7 (TLR7) is a pattern-recognition receptor that detects single-stranded viral RNA genomes and triggers an innate immune response [1]. Numerous inflammatory cells express TLR7, including T cells [2], B cells [3], eosinophils [4], and cells of the mononuclear phagocyte system such as macrophages and monocytes [5–8]. In these cells, endosomal-bound viral RNA activates TLR7, which initiates a Myd88-dependent signal transduction pathway leading to the secretion of type I interferons and proinflammatory cytokines that are essential for antiviral immunity [9].

Recently, non-canonical roles for TLR7 in both the central and peripheral nervous system have also been reported. In the central nervous system, intrathecal injection of TLR7 agonists caused axonal injury and neuronal cell death in mice in vivo [10, 11] and reduced dendrite growth in cultured mouse cortical neurons in vitro [12], suggesting TLR7 influences cortical neurogenesis. Intradermal injection of TLR7 agonists potentiated sensation of pain and itch in mice [13–15], suggesting TLR7 has neuromodulatory effects on peripheral sensory nerve function as well. Whether these effects are due to direct activation of neurons or mediated indirectly by second messengers released from TLR7-expressing support cells in ganglia, such as satellite glial cells and resident macrophages, is controversial. Previous studies reported conflicting results regarding TLR7 expression in ganglia, with some showing neuronal TLR7 expression using commercially available TLR7 antibodies, while others reported either the presence or absence of neuronal TLR7 RNA expression using RT-PCR [13–18]. These discordant findings have complicated efforts to develop a mechanistic understanding of TLR7 function in nervous tissues.

Our laboratory has been interested in interactions between TLR7 and peripheral nerves in the lungs given that RNA respiratory viruses potentiate nerve-mediated bronchoconstriction [19–23] and are a common trigger for asthma exacerbations in humans [24]. Thus, in previous studies, we tested whether the imidazoquinoline TLR7 agonists imiquimod (R837) and gardiquimod (R848) acutely increased nerve-mediated bronchoconstriction. On the contrary, we found that R837 and R848 paradoxically relaxed guinea pig airways in vivo and human airways ex vivo within minutes via production of nitric oxide [25, 26], suggesting TLR7 agonists have therapeutic potential as a novel class of bronchodilators. Based on this unexpected finding and since previous reports were contradictory regarding TLR7 expression by sensory nerves, we sought to characterize TLR7 expression within the vagal ganglia, which supply sensory innervation to the lungs, using in situ hybridization. RNA probe specificity was validated in TLR7 knockout (KO) tissues. We compared TLR7 expression in vagal ganglia to non-pulmonary sensory nerves within the dorsal root ganglia (DRG). The results from in situ experiments were compared with immunohistochemistry using two commonly-cited TLR7 antibodies from different commercial sources. Furthermore, we tested whether influenza A infection induces TLR7 expression in sensory ganglia and whether TLR7 agonists induce nitric oxide in neurons and support cells in ganglia.

## Methods

### Animals

Wild-type C57BL/6J and TLR7 KO (B6.129S1-*Tlr7*^*tm1Flv*^/J) mice were purchased from The Jackson Laboratory (Bar Harbor, ME) and were 11–18 weeks of age at the time of experimentation. Adult, female Hartley guinea pigs were purchased from Charles River Laboratories (Wilmington, MA) and were 7–10 weeks old (350–500 g) at the time of experimentation. All animals were housed in filtered air rooms, given ad libitum access to food and water, and exposed to a 12-h light/dark cycle. Animals were treated in accordance with the standards established by the United States Animal Welfare Act set forth by the National Institutes of Health guidelines. The Institutional Animal Care and Use Committee at Oregon Health & Science University approved all the experimental protocols.

### In situ hybridization

Mice and guinea pigs were euthanized with an overdose of pentobarbital. Animals were perfused with PBS, and tissues were dissected and fixed in 10% neutral buffered formalin for 16–24 h at room temperature (RT). Paraffin sections (5 μm) and tissue blocks were stored at 4 °C in a desiccator. The RNAscope in situ hybridization assay (Advanced Cell Diagnostics, San Francisco, CA) was performed per the manufacturer's instructions with a 15-min incubation in the RNAscope Target Retrieval reagent and a 30-min incubation in the RNAscope Protease Plus reagent (Advanced Cell Diagnostics, 322330). RNAscope probes (mouse TLR7 415411, mouse piptidylprolyl isomerase B (Ppib)-positive control 313911, guinea pig TLR7 563131, guinea pig Ppib-positive control 471531, dihyrdodipicolinate reductase (DapB)-negative control 310043, *E. coli* lacZ 538051, Advanced Cell Diagnostics) were detected with the RNAscope 2.5 HD Detection Reagents-RED kit (Advanced Cell Diagnostics, 322360). Slides were counterstained with Gills Hematoxylin (1:1 with water, American MasterTech Scientific) and imaged on a Zeiss Apotome Microscope (Oberkochen, Germany).

For in situ hybridization on cultured nodose and jugular ganglia, ganglia were dissociated from guinea pigs as previously described [27]. Cells were plated on laminin-coated (Life Technologies, Frederick, MD) glass-bottom fluorodishes (World Precision Instruments, Saratoga, FL) and cultured in media consisting of 1:1 F12:DMEM media (Thermo Fisher Scientific, Waltham, MA), 100 U/ml penicillin, 100 μg/ml streptomycin, 10 μg/ml guinea pig transferrin (BP25445, Fisher Scientific), 100 ng/ml nerve growth factor 2.5S (NG009, Sigma-Aldrich, St. Louis, MO), and 20 μM camptothecin (Sigma-Aldrich, St. Louis, MO). After 4 days, cells were fixed and processed for RNAscope in situ hybridization following the Advanced Cell Diagnostics Technical Note for Cultured Adherent Cells. Cells were incubated with guinea pig-specific probes for TLR7 RNA (Advanced Cell Diagnostics, guinea pig TLR7 563131), and the probes were detected with the RNAscope 2.5 HD Detection Reagents-RED (Advanced Cell Diagnostics, 322360) kit. Cells were coverslipped and imaged on a Nikon Eclipse Ci microscope (Tokyo, Japan) using an Andor Zyla camera at 100x (Nikon Plan Flour 1.3NA).

### Immunohistochemistry

Wild-type mice, TLR7 KO mice, and guinea pigs were euthanized with an overdose of pentobarbital and perfused with PBS. Dorsal root ganglia, vagal ganglia (containing the nodose and jugular ganglia), and spleens were collected for testing TLR7 in situ hybridization probes. Tissues were fixed in 10% neutral buffered formalin for 16–24 h at RT, then washed and prepared for paraffin sectioning. Slides were dewaxed with xylene and rehydrated with a decreasing series of alcohols to water.

For TLR7 immunohistochemistry, all slides were treated for 10 min in a 90 °C water bath with antigen unmasking solution (Vector Laboratories, Burlingame, CA) and then for 10 min in 3% hydrogen peroxide made in 4 °C methanol to block endogenous peroxidase. After washing, non-specific binding was blocked with 10% normal goat serum (Vector Laboratories) made in PBS containing 0.05% Tween-20 (PBST) for 1 h at RT. Slides were incubated overnight at 4 °C in a humidified chamber with either anti-TLR7 polyclonal antibody (1:1000, rabbit, Novus cat. # NBP2-24906) or anti-TLR7 monoclonal antibody (1:250, rabbit, Abcam cat. # ab124928) made in blocking buffer. A serial section did not receive a primary antibody to determine the background signal. The slides were then washed and incubated for 1 h at RT with a biotinylated secondary antibody (1:400, goat anti-rabbit, Vector Laboratories) and then 30 min with avidin biotin complex (Vector Laboratories). The slides were reacted with diaminobenzidine peroxidase (DAB, Vector Laboratories). Slides from TLR7 KO and wild type mice were reacted with DAB for the same duration. The slides were rinsed, counterstained with Gills Hematoxylin (1:1 with water, American MasterTech Scientific), mounted, and imaged on a Zeiss Apotome Microscope.

For ionized calcium-binding adaptor molecule 1 (Iba1), protein gene product 9.5 (PGP9.5), and glial fibrillary acidic protein (GFAP) immunohistochemistry following in situ hybridization, the slides were incubated in 3% hydrogen peroxide made in water for 10 min at RT to quench any remaining peroxidase activity from the in situ hybridization assay and then rinsed in water and PBST. To detect Iba1, a marker for macrophages/microglia, non-specific binding was blocked with 10% normal rabbit serum (Vector Laboratories) made in PBST for 1 h at RT, and then the slides were incubated with an anti-Iba1 antibody (1:2500, goat polyclonal, Abcam 5076, Cambridge, UK) overnight in a blocking buffer at 4 °C in a humidified chamber. The slides were then processed similarly as described above using a biotinylated rabbit anti-goat secondary antibody (1:400, Vector Laboratories). To detect PGP9.5, a pan-neuronal marker, non-specific binding was blocked with 10% normal goat serum (Vector Laboratories) made in PBST for 1 h at RT, and then slides were incubated with an anti-PGP9.5 antibody (1:1000, rabbit polyclonal, Millipore AB1761-I, Burlington, MA) overnight in blocking buffer at 4 °C in a humidified chamber. A goat anti-rabbit IgG H+L Alexa Flour 647 secondary antibody (1:1000, Thermo Fisher Scientific) was used for fluorescence detection of the antibody (1 h at RT) and then slides were mounted with Vectashield (Vector Laboratories) containing 4′,6-diamidino-2-phenylidndole (DAPI) to label the nuclei. To detect GFAP, a marker for satellite glial cells, slides were incubated overnight with anti-GFAP antibody (1:200, mouse IgG1, Neuromab 75-240, Davis, CA) at 4 °C in a humidified chamber, fixed in 10% neutral buffered formalin for 30 min at RT, and then TLR7 in situ hybridization was performed. A goat anti-mouse (H+L) biotinylated secondary antibody (1:200, Vector Laboratories) was incubated overnight at 4 °C, and slides were reacted with DAB. All slides were imaged on a Zeiss Apotome Microscope.

### Real-time reverse transcriptase-polymerase chain reaction (real-time RT-PCR)

Mouse vagal ganglia were aseptically dissected, and RNA was isolated using the RNeasy Mini Kit (Qiagen). cDNA was generated using Superscript III Reverse Transcriptase (Thermo Fisher Scientific) and amplified using a Veriti 96-well Thermal Cycler (Applied Biosystems, Foster City, CA). Mouse TLR7 primers (cat. # Mm00446590_m1) and 18s primers (cat. # 4352930E) were purchased from Applied Biosystems and amplified using TaqMan reagents by real-time RT-PCR (7500 Fast RT-PCR system, Applied Biosystems). TLR7 expression was normalized using delta-delta CT.

### Quantification of cellular nitric oxide

Neurons and support cells from dissociated ganglia were plated on laminin-coated (Life Technologies, Frederick, MD) glass-bottom fluorodishes (World Precision Instruments, Saratoga, FL) and cultured in media consisting of 1:1 F12:DMEM media (Thermo Fisher Scientific, Waltham, MA), 100 U/ml penicillin, 100 μg/ml streptomycin, 10 μg/ml guinea pig transferrin (BP25445, FisherScientific), 100 ng/ml nerve growth factor 2.5S (NG009, Sigma-Aldrich, St. Louis, MO), and 20 μM camptothecin (Sigma-Aldrich, St. Louis, MO) for 48 h. Cells were loaded with a nitric oxide-detecting fluorophore (FL2A, Strem Chemicals) for 30 min followed by treatment with TLR7 agonist R837 (10 μM, Invitrogen) for an additional 30 min. Some cells were pre-treated with TLR7 antagonist IRS661 (100 μM, Integrated DNA Technologies), TLR7 antagonist control oligomer (100 μM, Integrated DNA Technologies), or the nitric oxide synthase antagonist L-NAME (100 μM, Sigma). Cells were imaged on a Nikon spinning-disk confocal microscope (× 20, 1.3 NA), and cellular fluorescence was quantified within individual cell bodies using ImageJ. Experimental replicates represent the average of 10–20 individual cells per experimental condition.

### H1N1 infection

Wild-type mice were anesthetized with ketamine (45 mg/kg) and xylazine (8 mg/kg) i.p. and infected by intranasal administration (25 μl) of influenza A virus subtype H1N1 (ATCC A/PR/8/34 strain, 1e^5^ TCID_50_ units) or mock-infected with PBS vehicle. Viral stocks were grown in rhesus monkey kidney (RMK) cell monolayers, purified by sucrose density centrifugation, and titered in RMK cells. Mice were euthanized by an overdose of pentobarbital (150 mg/kg i.p.) 4 days later. The right lung and left vagal ganglia were flash-frozen for RNA isolation using the RNeasy Mini Kit (Qiagen). The right vagal ganglia and spleen were fixed in 10% neutral buffered formalin at RT for 16–24 h for in situ hybridization assays.

### Quantification of H1N1 RNA in lung

Primers for H1N1 were synthesized by Integrated DNA Technologies (Coralville, IA) as follows: 5′ CATCCTGTTGTATATGAGGCCCAT and 3′ TTCGCAGATGCGACGTCAGG. H1N1 titers from the whole lung were quantified by real-time RT-PCR (7500 Fast RT-PCR system, Applied Biosystems) using an H1N1 TCID_50_ standard curve derived from serial dilutions of cDNA generated from the H1N1 viral stock solution. Samples were normalized for 18S cDNA.

### Analysis of RNAscope in situ hybridization

Images of H1N1-infected or mock-infected mouse spleen and vagal ganglia labeled with RNAscope in situ probes for TLR7 or Ppib (housekeeping gene) were imaged at  60x on a Zeiss Apotome Microscope. The same light intensity and exposure time were used to image H1N1- and mock-infected tissues, but varied between the different tissues and probes to obtain optimal images for each tissue and probe. For the spleen, a 60x image was taken from 3 different portions from the same mouse, and for the vagal ganglia, multiple 60x images capturing the whole ganglia within a single section were obtained for each animal. Images were analyzed by ImageJ (version 1.52p, National Institutes of Health), where a color threshold was set to detect the signal by the in situ probes. Each image was closely reviewed to ensure that the area detected by the set threshold and included in the analysis was a true signal from an in situ probe. The area of TLR7 expression within 3 different pieces of spleen was normalized to the area of Ppib expression in a similar location in a serial section, to normalize for differences in RNA integrity between animals. For the vagal ganglia in each mouse, the total area of RNAscope signal was normalized to the total area of vagal ganglia analyzed, which included only the ganglia and not large nerve fibers passing through. Again, TLR7 expression in the vagal ganglia was normalized to Ppib expression detected in the same region of a serial section to control for the differences in RNA integrity between animals.

### Statistics

Comparison of mouse weights before and after infection, and cellular nitric oxide fluorescence, was analyzed by a one-way ANOVA with a Sidak post hoc test for multiple comparisons. Quantification of viral titers in the lungs and in situ hybridization labeling in the spleen and vagal ganglia of mock- and H1N1-infected mice were analyzed by a two-tailed unpaired *t*-test. All data was analyzed using GraphPad Prism (8.3.0, San Diego, CA). *p* values less than 0.05 were considered significant.

## Results

### TLR7 is expressed by non-neuronal cells in dorsal root and vagal ganglia

**Fig. 1:**
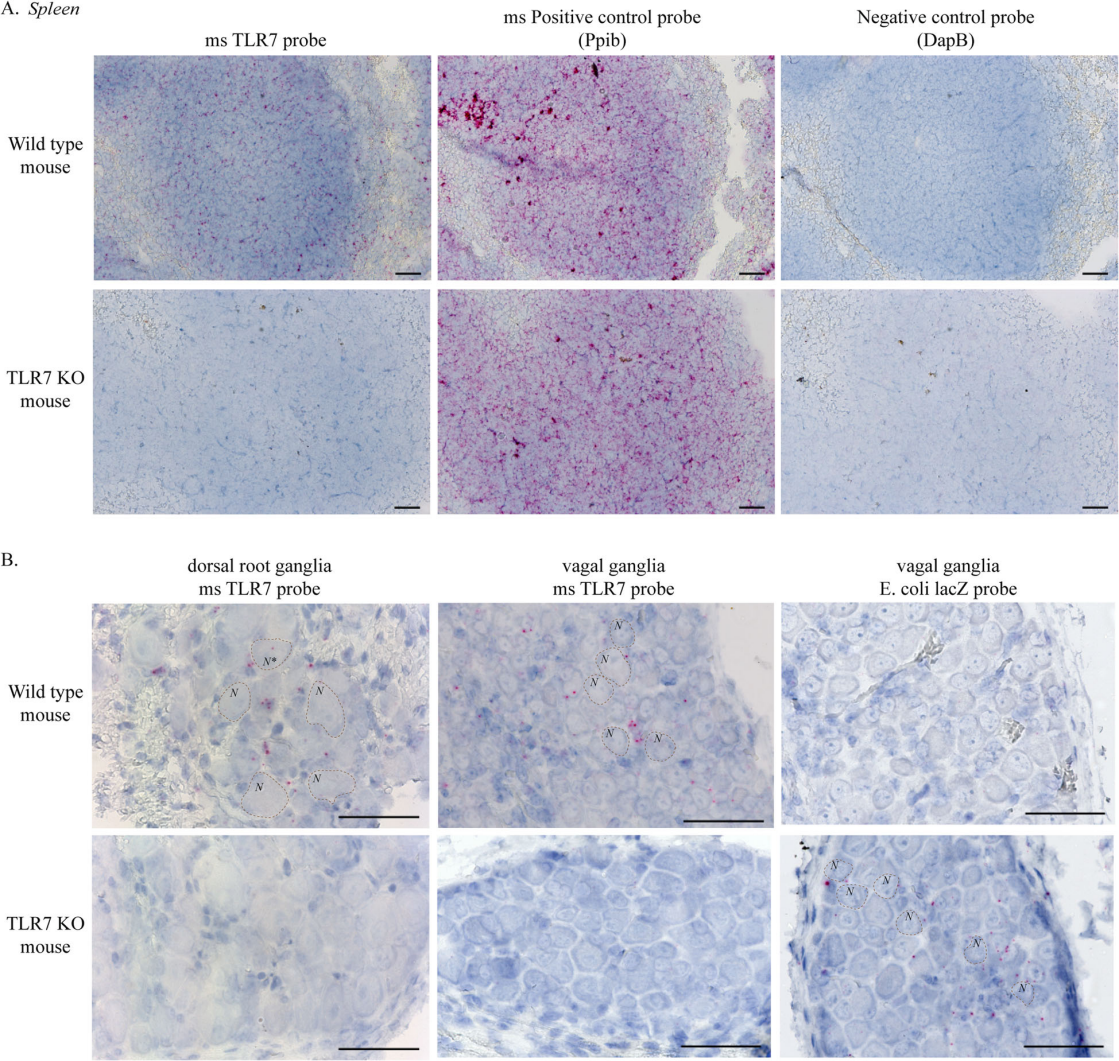
TLR7 is expressed by peri-neuronal support cells in mouse sensory ganglia. **A** Mouse-specific TLR7 in situ hybridization probe was tested in wild-type and TLR7 KO mouse spleen. TLR7 (pink) was detected in the spleen from wild-type mice, but not in TLR7 KO mice, confirming the specificity of the probe. The housekeeping gene Ppib (pink) was used as a positive control to confirm tissue permeability and RNA integrity, and an in situ probe for the bacterial protein DapB served as a negative control to assess the in situ hybridization background signal. **B** TLR7 in situ probes labeled small cells located in close proximity to neuronal cell bodies (*N*) within dorsal root ganglia and vagal ganglia in wild-type mice. Rarely, a single TLR7 in situ probe signal could be found in a neuronal cell body (N*). TLR7 was absent in both dorsal root and vagal ganglia from TLR7 KO mice. Vagal ganglia were also labeled with in situ hybridization probes targeting lacZ since TLR7 KO mice were generated by inserting an *E. coli* lacZ gene into exon 3 of the mouse TLR7 gene. LacZ in situ probes labeled small support cells, but not neurons in vagal ganglia from TLR7 KO mice. Blue = hematoxylin nuclear stain in all images. *N* = 4, representative images are shown. Scale bar = 50 μm

The specificity of RNA-specific mouse TLR7 probes was assessed in wild-type and TLR7 KO mice using in situ hybridization. As per previous reports [28], TLR7 was extensively expressed in wild-type mouse spleens (Fig. 1A). TLR7 was not detected in the spleens from TLR7 KO mice, indicating that the probe was specific for TLR7 mRNA. The expression of the housekeeping gene piptidylprolyl isomerase B (Ppib), a cyclosporine-binding protein, was detected in serial sections of both the wild-type and TLR7 KO mouse spleen (Fig. 1A), confirming that RNA was present in all the tissue. A negative control probe for the bacterial gene dihyrdodipicolinate reductase (DapB) showed no signal in either the wild-type or TLR7 KO spleen (Fig. 1A). TLR7 expression was also detected in the dorsal root (Fig. 1B, left panel) and vagal ganglia (Fig. 1B, middle panel) from wild-type mice and specifically localized to small cells alongside nerve cell bodies, but not within the nerves. In rare instances, a single TLR7 in situ signal was observed within a neuronal cell body (Fig. 1B, neuron labeled N*), indicating either that neuronal TLR7 expression was exceptionally uncommon or that an adjacent TLR7-expressing support cell overlapped a neuron in the *Z* plane of the tissue section. TLR7 was absent in the dorsal root and vagal ganglia from TLR7 KO mice (Fig. 1B). Control experiments confirming positive expression of the housekeeping gene Ppib and lack of DapB expression were performed in parallel in serial sections from wild-type and TLR7 KO mouse dorsal root ganglia (Supplemental Figure 1A) and vagal ganglia (Supplemental Figure 1B).

TLR7 KO mice were generated by inserting an *E. coli* lacZ gene into exon 3 of the mouse TLR7 gene. Thus, lacZ expression was tested in wild-type and TLR7 KO mice using an *E. coli* lacZ RNA probe. LacZ was present in peri-neuronal support cells in the vagal ganglia, but was absent in the vagal sensory neurons in TLR7 KO mice (Fig. 1B, right panel). LacZ expression was not detected in wild-type mice.

**Fig. 2:**
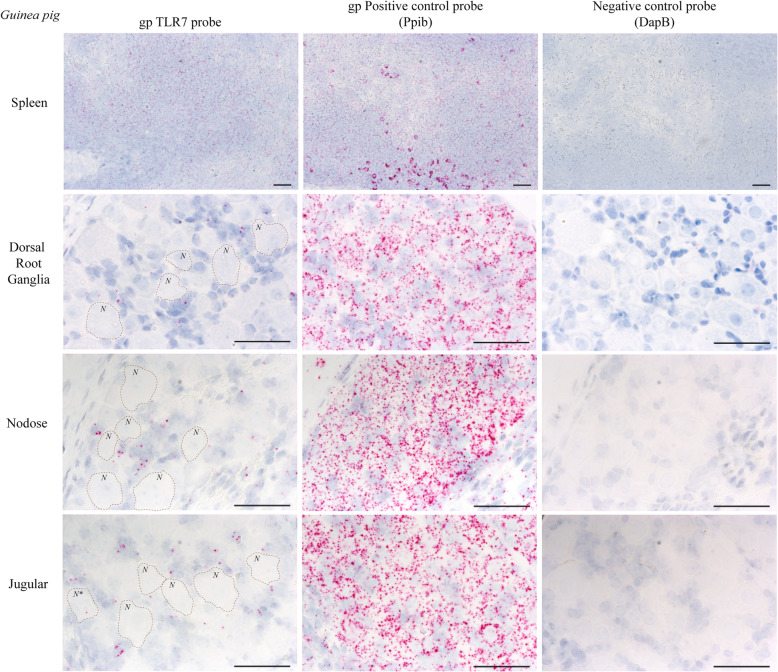
TLR7 is expressed by support cells in guinea pig vagal and dorsal root ganglia. Guinea pig-specific in situ hybridization probe detected TLR7 (pink) in small cells located outside the neuronal cell bodies (*N*) in the dorsal root ganglia and in the nodose and jugular ganglia, which comprise the vagal ganglia. The housekeeping gene Ppib (pink) was also detected in the spleen and ganglia, while the negative control probe (DapB) showed no signal as expected. Rarely, a single TLR7 in situ probe signal could be found in a neuronal cell body (N*). Representative neuronal cell bodies (*N*) within the ganglia are outlined in the image. Blue = hematoxylin nuclear stain in all images. *N* = 4, representative images are shown. Scale bar = 50 μm

A separate, guinea pig-specific RNA probe for TLR7 similarly detected TLR7 in guinea pig spleen (Fig. 2) and in small cells located adjacent to nerve cell bodies in the nodose, jugular, and dorsal root ganglia. As expected, a guinea pig-specific probe for Ppib detected RNA in serial sections throughout the ganglia, including nerve cell bodies, and the negative control probe DapB was not detected, confirming that the TLR7 probe signal was not the result of the background signal (Fig. 2).

### TLR7 is expressed by Iba1-positive macrophages and GFAP-positive satellite glial cells in ganglia

**Fig. 3:**
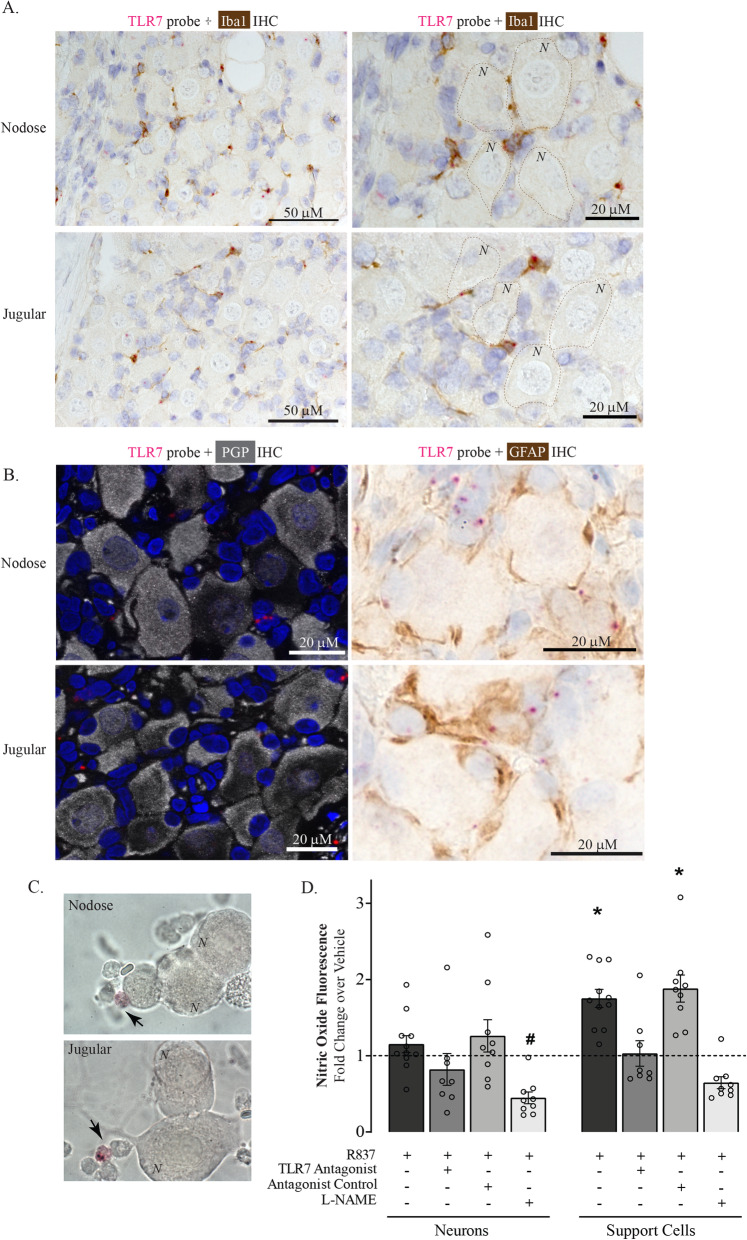
TLR7 is co-expressed in Iba1- and GFAP-expressing cells within the nodose and jugular ganglia. Guinea pig nodose and jugular ganglia were co-labeled with TLR7 in situ hybridization probes and antibodies for colorimetric and fluorescent immunohistochemistry. **A** TLR7 RNA (pink) colocalized with cells expressing the macrophage marker Iba1 (brown). Blue = hematoxylin or DAPI nuclear stain. Representative images are shown, *N* = 4. *N* = neurons (selected neurons are labeled in the images). **B** TLR7 was not expressed in the neurons labeled with the pan-neuronal marker PGP9.5 (gray, left panels), but was expressed in GFAP-positive satellite glial cells (brown, right panels). **C** TLR7 in situ probes detected TLR7 (pink, arrows) in small cells from dissociated nodose and jugular ganglia. *N* = neurons. **D** Cultured neurons and support cells from dissociated ganglia were loaded with the nitric oxide detecting fluorophore and treated with the TLR7 agonist R837. Support cells, but not neurons, produced nitric oxide in response to a TLR7 agonist. *N* = 8–10 replicates. Each replicate represents the average from 10 to 20 individual cells

Guinea pig nodose and jugular ganglia sections were co-labeled with antibodies against the macrophage marker Iba-1, against the satellite glial cell marker GFAP, and against the neuronal protein PGP9.5 after performing TLR7 in situ hybridization. TLR7 colocalized with Iba1-expressing cells (Fig. 3A) and separately with GFAP-expressing cells outside and in close proximity to neuronal cell bodies within both the nodose and jugular ganglia (Fig. 3B). TLR7 RNA expression did not overlap with the neuronal marker PGP9.5 (Fig. 3B). Similarly, TLR7 was expressed by small support cells, but not neurons in cultures of dissociated vagal ganglia (Fig. 3C). These data indicate TLR7 is not expressed in sensory nerve cell bodies, but instead is expressed in non-neuronal macrophage and satellite glial support cells within the ganglia.

### Support cells, but not neurons, produce nitric oxide in response to TLR7 agonist

Dissociated neurons and support cells were loaded with a nitric oxide detecting fluorophore and treated with a TLR7 agonist R837 in culture. Support cells, but not neurons, increased their nitric oxide fluorescence in response to TLR7 agonist (Fig. 3D). TLR7-mediated nitric oxide release from support cells was blocked by TLR7 antagonist and by the nitric oxide synthase inhibitor L-NAME.

### TLR7 expression increases during influenza A infection in mouse spleen, but not vagal ganglia

**Fig. 4:**
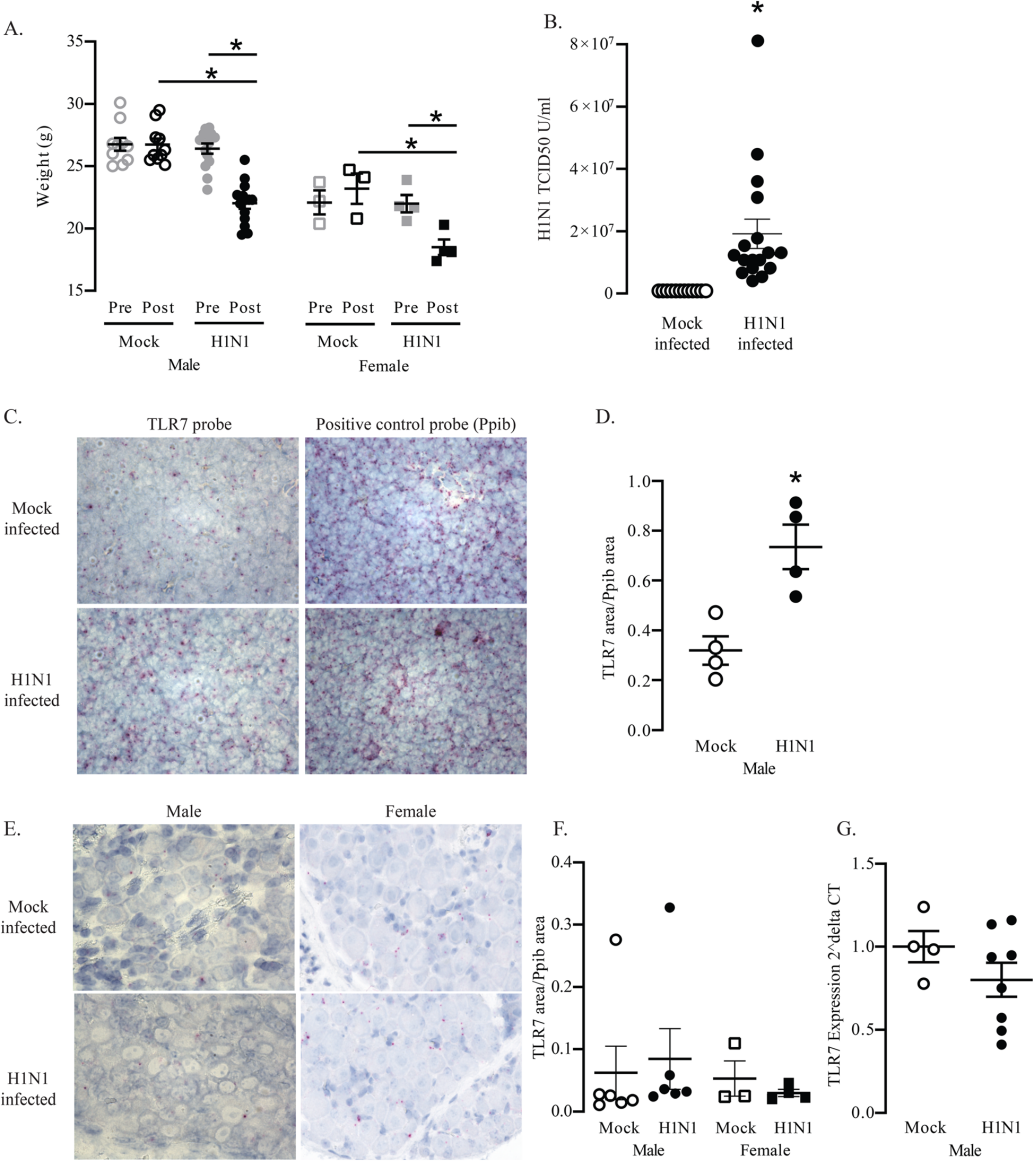
TLR7 RNA expression in mouse spleen and vagal ganglia after H1N1 infection. **A** Wild-type mice were weighed before (pre) and 4 days after (post) inoculation with H1N1 (H1N1-infected) or PBS vehicle (mock-infected). Body weights were significantly reduced in H1N1-infected mice. **B** H1N1 viral titers were quantified from lung tissue by real-time RT-PCR 4 days after inoculation. H1N1 viral RNA was present only in the mice inoculated with H1N1. *N* = 12–17. **C** Sections of the spleen were labeled with either the TLR7 or the housekeeping gene Ppib in situ hybridization probes (pink, representative images). Blue = hematoxylin nuclear stain. **D** TLR7 RNA was quantified and normalized to housekeeping RNA Ppib expression. H1N1 infection significantly increased the TLR7 RNA expression in mouse spleen. **E** Sections of the vagal ganglia were labeled with either TLR7 or the housekeeping gene Ppib in situ hybridization probes (pink, representative images). Blue = hematoxylin nuclear stain. **F** TLR7 RNA expression was quantified and normalized to the Ppib expression. H1N1 infection did not affect the TLR7 expression in mice vagal ganglia. **G** Similarly, in the contralateral ganglia, TLR7 RNA expression, quantified by real-time RT-PCR, was not affected by H1N1 infection. *p < 0.05

Wild-type mice were inoculated with H1N1 influenza A or vehicle to determine if respiratory virus infection induces TLR7 expression, particularly in the vagal neurons. Four days after inoculation, H1N1-infected mice lost significant weight (Fig. 4A) and H1N1 RNA was detected in their lung homogenates using real-time RT-PCR (Fig. 4B). Viral RNA was not present in mock-infected animals. In H1N1-infected animals, TLR7 RNA expression was significantly increased in the spleen 4 days after infection (Fig. 4C, D). In contrast, H1N1 infection did not increase TLR7 expression in the vagal ganglia overall (Fig. 4E, F), nor did viral infection induce de novo TLR7 expression in the nerve cell bodies (Fig. 4E). For further confirmation, the contralateral vagal ganglia was homogenized and analyzed for TLR7 RNA expression by quantitative RT-PCR. Similar to the result obtained by the in situ hybridization assay, TLR7 RNA expression was not increased in the vagal ganglia from H1N1-infected mice (Fig. 4G). These data show that TLR7 RNA expression in the vagal ganglia is not affected by H1N1 infection.

### Commercial TLR7 antibodies label small and medium sensory neurons in a TLR7-independent manner

**Fig. 5:**
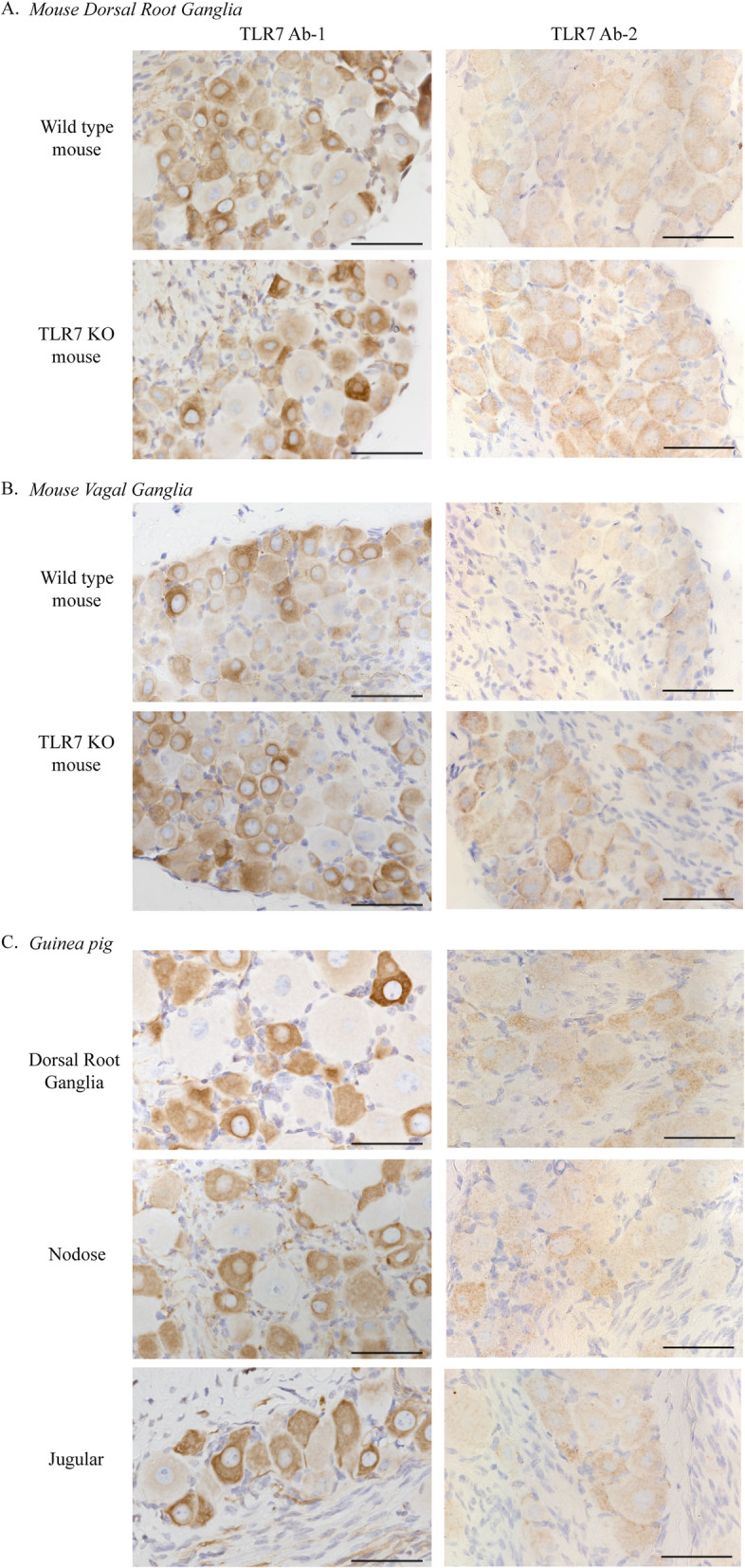
Immunohistochemistry with TLR7 antibodies reveals TLR-independent labeling of small- and medium-sized sensory neurons. Mouse dorsal root ganglia and vagal ganglia were collected from wild-type mice and TLR7 KO mice, and paraffin sections were processed for immunohistochemistry. Both TLR7 antibodies (TLR7 Ab-1, Novus; TLR7 Ab-2, Abcam) labeled small-to-medium-sized neurons (brown) in **A** dorsal root ganglia and **B** vagal ganglia in both wild-type and TLR7 KO mice, indicating that this antibody is not specific for TLR7. **C** An identical staining pattern for TLR7 (brown) was detected in guinea pig dorsal root ganglia and nodose and jugular ganglia. Scale bar = 50 μm. Blue = hematoxylin nuclear stain. *N* = 4, representative images are shown

Sections of wild-type, TLR7 KO, and guinea pig dorsal root ganglia and vagal ganglia were immunolabeled with previously cited commercial TLR7 antibodies [13, 15, 29–31]. Tissue sections from wild-type and TLR7 KO mice were processed simultaneously and reacted with DAB for the same duration to ensure equal treatment. In both wild-type and TLR7 KO mice, TLR7 antibodies labeled small- to medium-sized neurons within the dorsal root ganglia (Fig. 5A and Supplemental Figure 2) and nodose and jugular ganglia (Fig. 5B and Supplemental Figure 2). In general, the TLR7 antibody did not label larger neurons in either the dorsal root ganglia or vagal ganglia from both the wild-type and TLR KO mice. This pattern of staining was also observed in guinea pig dorsal root ganglia and nodose and jugular ganglia (Fig. 5C and Supplemental Figure 2). Slides that did not receive primary antibody were processed in parallel with other slides and showed no background labeling from secondary antibodies alone (Supplemental Figure 2). Thus, although the pattern of TLR7 antibody staining appears to indicate TLR7 expression in small and medium sized sensory neurons, staining in both wild type and in TLR7 KO mice clearly demonstrates that these antibodies are not specific for TLR7.

## Discussion

Determining TLR7’s neuromodulatory mechanisms in sensory ganglia has been complicated by conflicting reports indicating both the presence and absence of TLR7 expression by sensory nerves and by off-target effects of some TLR7 agonists [13–18, 32]. As a result, whether TLR7’s effects on nerve function are due to direct activation of nerves or mediated by secondary messengers released from support cells in ganglia, such as macrophages and satellite glial cells, is not clear. Several studies that show neuronal TLR7 expression have relied solely on antibody-based immunohistochemistry [13, 15, 29, 30] or have analyzed expression using PCR products from dissociated ganglia consisting of both neurons and non-neuronal support cells [13, 30]. Here, we clearly show that peri-neuronal support cells, but not sensory neurons, express TLR7 in the vagal and dorsal root ganglia. We also show using immunohistochemistry that previously cited TLR7 antibodies label small- and medium-sized neurons in a TLR7-independent manner. These findings suggest TLR7 agonists modulate peripheral sensory nerve function in the ganglia indirectly, likely through the activation of TLR7-expressing macrophages and satellite glial cells. Our results also indicate that other reports of neuronal TLR7 expression on enteric nerves [30] and trigeminal ganglia [29, 31], which were based on immunohistochemistry, should be re-examined.

Ganglia support cells, of which macrophages and satellite glial cells are some of the most common, have a key role in sensory nerve homeostasis and modulate neuronal excitability in the setting of nerve injury [33, 34]. As in other tissues, macrophages in ganglia are phagocytic cells that contribute to antigen-presentation [35, 36], whereas satellite glial cells are unique to peripheral nervous tissues, providing structural support by closely surrounding neuronal somata and directly communicating with other satellite glial cells via gap junctions [34]. Following nerve injury, neuronal mediators including nitric oxide and ATP activate macrophages and satellite glial cells [37, 38], and in turn, support cells contribute to nerve regeneration and repair [39, 40]. Activated support cells also release inflammatory cytokines TNF-alpha and IL-1beta [38], which sensitize neuronal nociceptors and increase nerve excitability to acutely enhance sensations of pain [33, 41]. Due to unknown factors in some individuals, nociceptor sensitization persists and results in disorders of chronic pain and chronic itch [42]. The factors that bias support cells and their mediators towards the development of these pathologic nerve states is an area of intense interest given their potential as therapeutic targets in these conditions.

TLR7 stimulation on macrophages and satellite glial cells [43, 44] may contribute to sensory nerve sensitization. TLR7 agonists potentiated chloroquine-induced, but not histamine-induced scratching behavior in mice, while TLR7-deficient mice had reduced scratching, suggesting TLR7 mediates pruritis by increasing peripheral sensory nerve excitability in response to non-histaminergic stimuli [15]. Similarly, TLR7 activation enhanced withdrawal responses to painful stimuli in mice [13, 14, 16]. In both instances, direct activation of TLR7 on sensory nerves was proposed. However, our results suggest direct activation of TLR7 on neurons is unlikely since TLR7 expression was essentially absent from sensory nerve cell bodies. Only rarely was a single TLR7 in situ signal localized in a nerve, and since this signal is not robust, it is highly unlikely that neurons express TLR7. Several reasons may account for discrepancies between our results and previous studies. For one, we show that the pattern of anti-TLR7 antibody staining in small- to medium-sized sensory neurons in both vagal and dorsal root ganglia, which previous studies have cited as evidence of neuronal TLR7 expression, is in fact binding a target other than TLR7 since staining was identical in both wild type and TLR7 KO mice. In contrast, TLR7 situ hybridization probes show TLR7-specific staining in peri-neuronal support cells in wild-type, but not in TLR7 KO, mice and do not show staining in neurons in either wild-type or TLR7 KO mice. The TLR7 RNA probe additionally labeled cells in the spleen from wild-type, but not TLR7 KO, mice, providing further evidence of TLR7 probe specificity. Secondly, in situ probes against lacZ inserted in the TLR7 gene in TLR7 KO mice show a similar peri-neuronal pattern for lacZ in ganglia support cells, but not within neurons in TLR7 KO mice. Thirdly, previous RT-PCR-based analyses tested RNA from the whole ganglia and often pooled multiple ganglia together to obtain sufficient RNA, which contain a variety of non-neuronal TLR7-expressing support cells. Fourthly, in many cases, the depolarizing effects of TLR7 imidazoquinoline agonists in single neurons also have well-established TLR7-independent effects on intracellular calcium, particularly at concentrations reported to induce itch [32, 45]. Finally, it is unlikely that strain- and species-specific differences account for differing results since our study and most prior studies both used mice on a C57BL/6 background and since our results using TLR7-specific RNA probes were reproducible in guinea pigs as well as mice.

In both neuronal and non-neuronal tissues, TLR7 is activated by GU-rich single-stranded RNA [1, 14]. However, unlike in other tissues, TLR7’s role in the nervous system may not be primarily for antiviral immunity. For example, TLR7 was not required for viral clearance from the central nervous system in mice infected with polytropic retrovirus Fr98 despite producing an abundance of neuroinflammatory mediators including interferon gamma, TNF-alpha, and CCL2 [46]. The absence of TLR7 in KO mice also had no effect on viral replication. Our results similarly do not suggest TLR7 in sensory ganglia has a role during influenza A infection given that TLR7 expression did not increase in ganglia support cells nor was TLR7 expression induced in neurons de novo 4 days after inoculation. While our results do not definitively exclude a role for TLR7 in ganglia during infection, since functional changes in the TLR7 receptor, its downstream signaling mediators, or chaperone proteins involved in TLR7 trafficking in lysosomes may occur independent of RNA expression, it is notable that TLR7 in the spleen was significantly upregulated by influenza A suggesting that alterations in TLR7 transcription would be observed in ganglia if TLR7’s role in antiviral defense was similar in both tissues.

The full scope of TLR7’s functions in the nervous system remains an open question. TLR7 has recently been shown to serve as a receptor for the endogenous microRNA Let-7b, which may help regulate microglial pruning of neurons in the central nervous system during early development in humans [47] and is elevated in neurodegenerative diseases such as Alzheimer’s [48] where it may contribute to neuronal loss [11, 12]. Japanese encephalitis virus provoked Let7b release from neurons, causing microglial caspase activation leading to neuronal cell death [49]. In the peripheral nervous system, activated dorsal root sensory nerves released Let-7b in vitro and intraplantar injections of Let-7b induced pain in mice [14], suggesting microRNA’s act as paracrine signals via TLR7 within sensory ganglia as well.

Our laboratory has focused on TLR7’s role in pulmonary afferents given that influenza A, parainfluenza, and other respiratory viruses, which bind to TLR7, also potentiate nerve-mediated bronchoconstriction over a matter of days [19–22], and since synthetic TLR7 agonists paradoxically relax airways within minutes via release of nitric oxide [25]. TLR7 expression was also reportedly increased in severe asthma [50]. Our current results effectively excludes activation of TLR7 directly on pulmonary sensory nerves by viruses or by synthetic agonists. Whether these ligands influence nerve function indirectly via TLR7 activation on support cells, possibly via support cell release of nitric oxide [37], is the focus of future investigations.

## Conclusion

Using species-specific TLR7 probes for in situ hybridization, we show that TLR7 is not expressed in the neurons of sensory ganglia as previously suggested, but instead in ganglia support cells including macrophages and satellite glial cells that are in close proximity to neurons. Thus, TLR7’s neuromodulatory effects on peripheral sensory nerves are likely mediated indirectly through ganglia support cells. Our results also do not support a role for TLR7 in ganglia in the immune response to respiratory viral infection. TLR7’s expression and function in ganglia are important considerations when designing TLR7-targeted therapeutics to treat pathologic nerve states like chronic pain.

## Supplementary Information

**Additional file 1: Figure S1.** In situ hybridization positive and negative controls for mouse dorsal root and vagal ganglia. Expression of the housekeeping gene piptidylprolyl isomerase B (Ppib), a cyclosporine-binding protein, was detected in serial sections of wild type and TLR7 knockout (KO) mouse (**A**) dorsal root ganglia and **(B)** vagal ganglia, confirming that RNA was present all the tissue. A negative control probe for the bacterial gene dihyrdodipicolinate reductase (DapB) was not detected in wild type or TLR7 KO (**A**) dorsal root ganglia and **(B)** vagal ganglia, excluding the presence of non-specific probe binding. Scale bar = 50 μm. Blue = hematoxylin nuclear stain.

**Additional file 2: Figure S2.** Immunohistochemistry with TLR7 antibodies indicates TLR-independent labeling of sensory neurons. Wild type and TLR7 knock out (KO) mouse dorsal root ganglia and vagal ganglia were paraffin embedded and processed for immunohistochemistry. Two separate TLR7 antibodies (TLR7 Ab-1, Novus; TLR7 Ab-2, Abcam) labeled small-to-medium-sized neurons (brown) in **(A)** dorsal root ganglia and **(B)** vagal ganglia in both wild type and TLR7 KO mice, and in **(C)** guinea pig dorsal root, nodose, and jugular ganglia. No primary antibody control experiments performed in parallel with TLR7 staining excluded non-specific secondary staining as the cause for neuronal labeling. Scale bar = 50 μm. Blue = hematoxylin nuclear stain. N = 4, representative images shown.

## Data Availability

The datasets generated and analyzed for this study are available from the corresponding author on reasonable request.

## Abbreviations

DapB: Dihyrdodipicolinate reductase
DAPI: 4′,6-Diamidino-2-phenylidndole
GFAP: Glial fibrillary acidic protein
Iba1: Ionized calcium-binding adaptor molecule 1
KO: Knockout
PBST: Phosphate-buffered saline containing 0.05% Tween-20
PGP9.5: Protein gene product 9.5
Ppib: Piptidylprolyl isomerase B
RT: Room temperature
RT-PCR: Reverse transcriptase polymerase chain reaction
TCID_50_: Mean tissue culture infectious dose
TLR7: Toll-like receptor 7
