# Supplementary figures and images for: TLR7 is expressed by support cells, but not sensory neurons, in ganglia
Source: J Neuroinflammation. 2021 Sep 16;18:209. doi: 10.1186/s12974-021-02269-x (PMC8447680; doi:10.1186/s12974-021-02269-x)

## Supplemental Figure S1

### A. *Dorsal Root Ganglia*

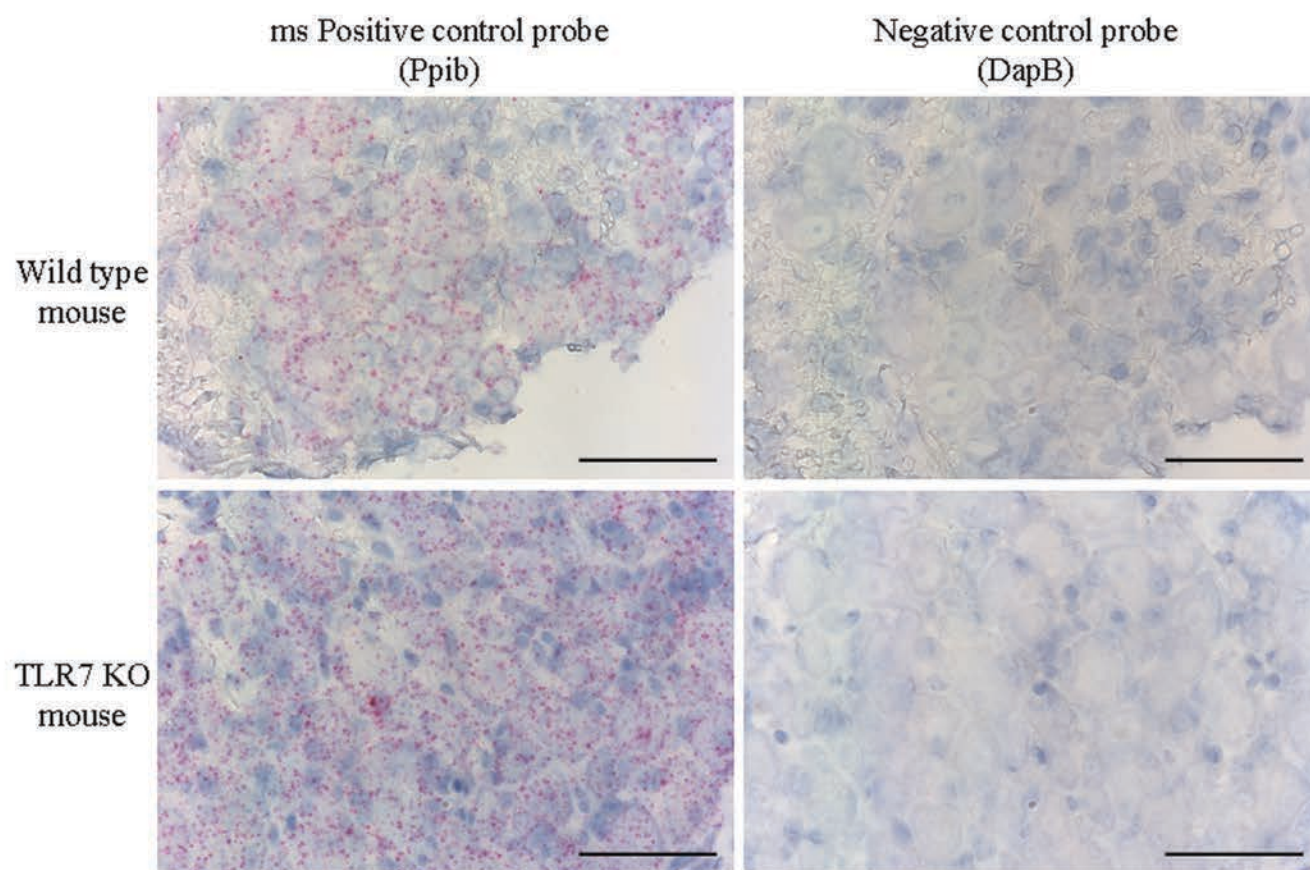

### B. *Vagal Ganglia*

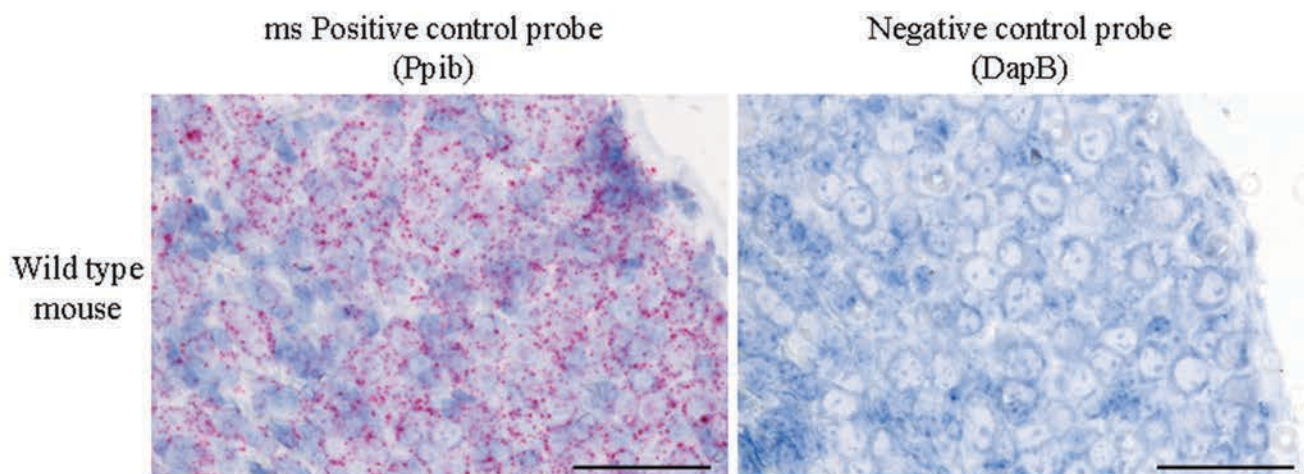

Supplement: Supplementary file 1 — Additional file 1: Figure S1. In situ hybridization positive and negative controls for mouse dorsal root and vagal ganglia. Expression of the housekeeping gene piptidylprolyl isomerase B (Ppib), a cyclosporine-binding protein, was detected in serial sections of wild type and TLR7 knockout (KO) mouse (A) dorsal root ganglia and (B) vagal ganglia, confirming that RNA was present all the tissue. A negative control probe for the bacterial gene dihyrdodipicolinate reductase (DapB) was not detected in wild type or TLR7 KO (A) dorsal root ganglia and (B) vagal ganglia, excluding the presence of non-specific probe binding. Scale bar = 50 μm. Blue = hematoxylin nuclear stain. [file 12974_2021_2269_MOESM1_ESM.pdf]

Supplemental Figure S2.

A. *Mouse Dorsal Root Ganglia*

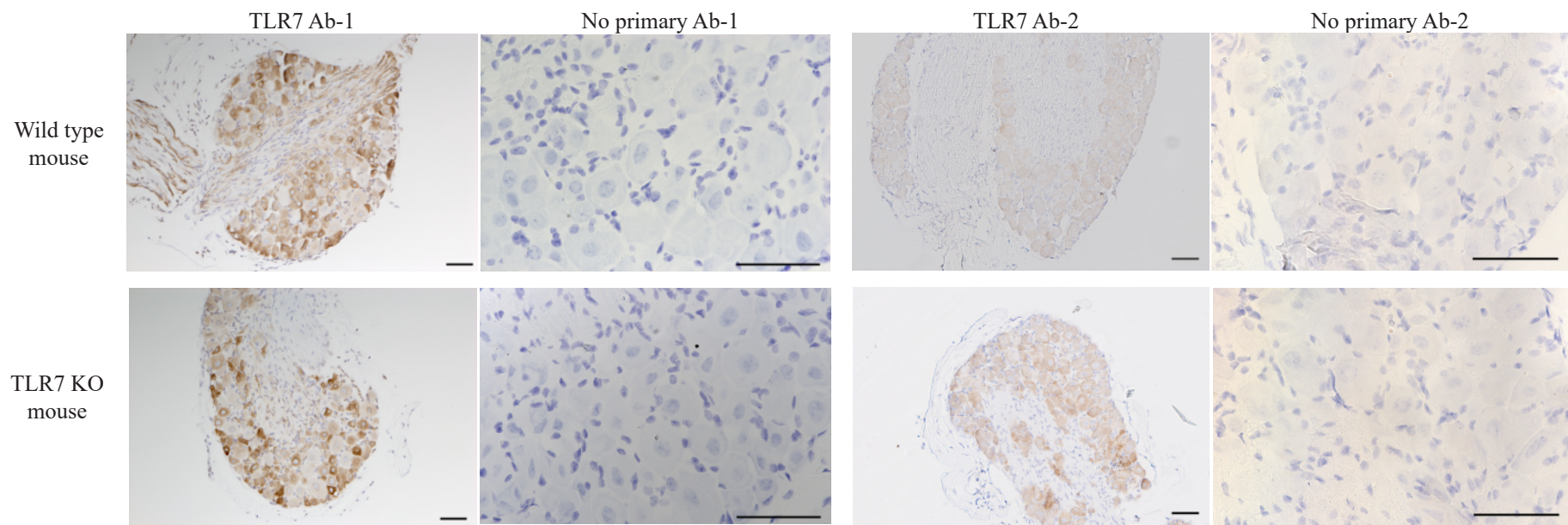

B. *Mouse Vagal Ganglia*

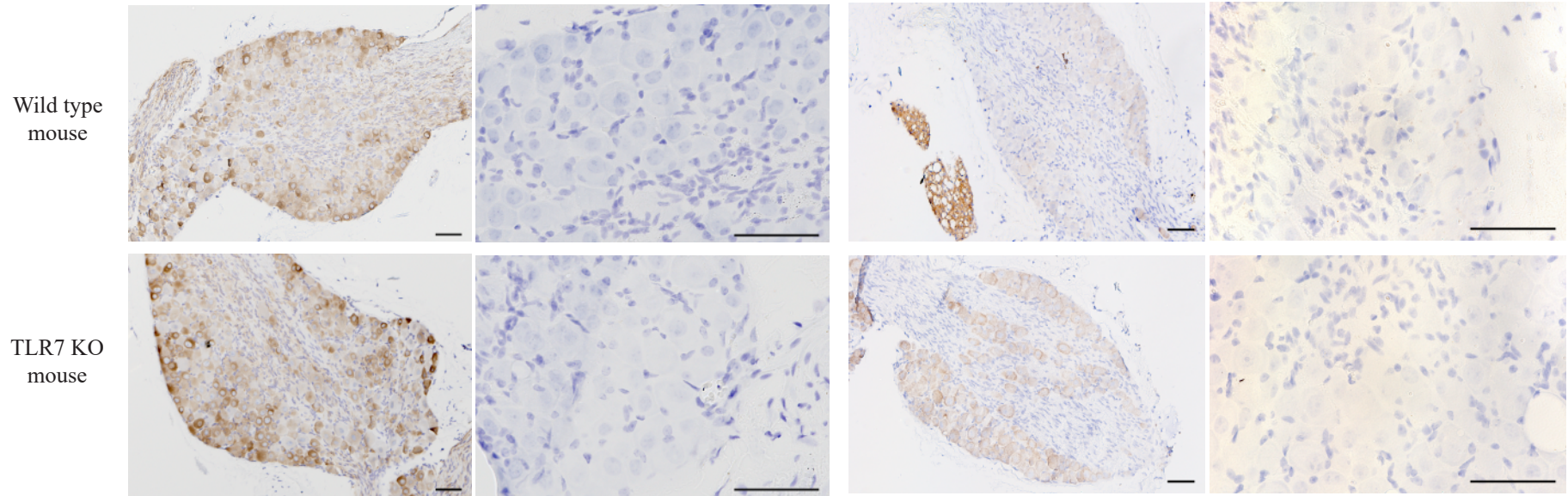

C. *Guinea pig*

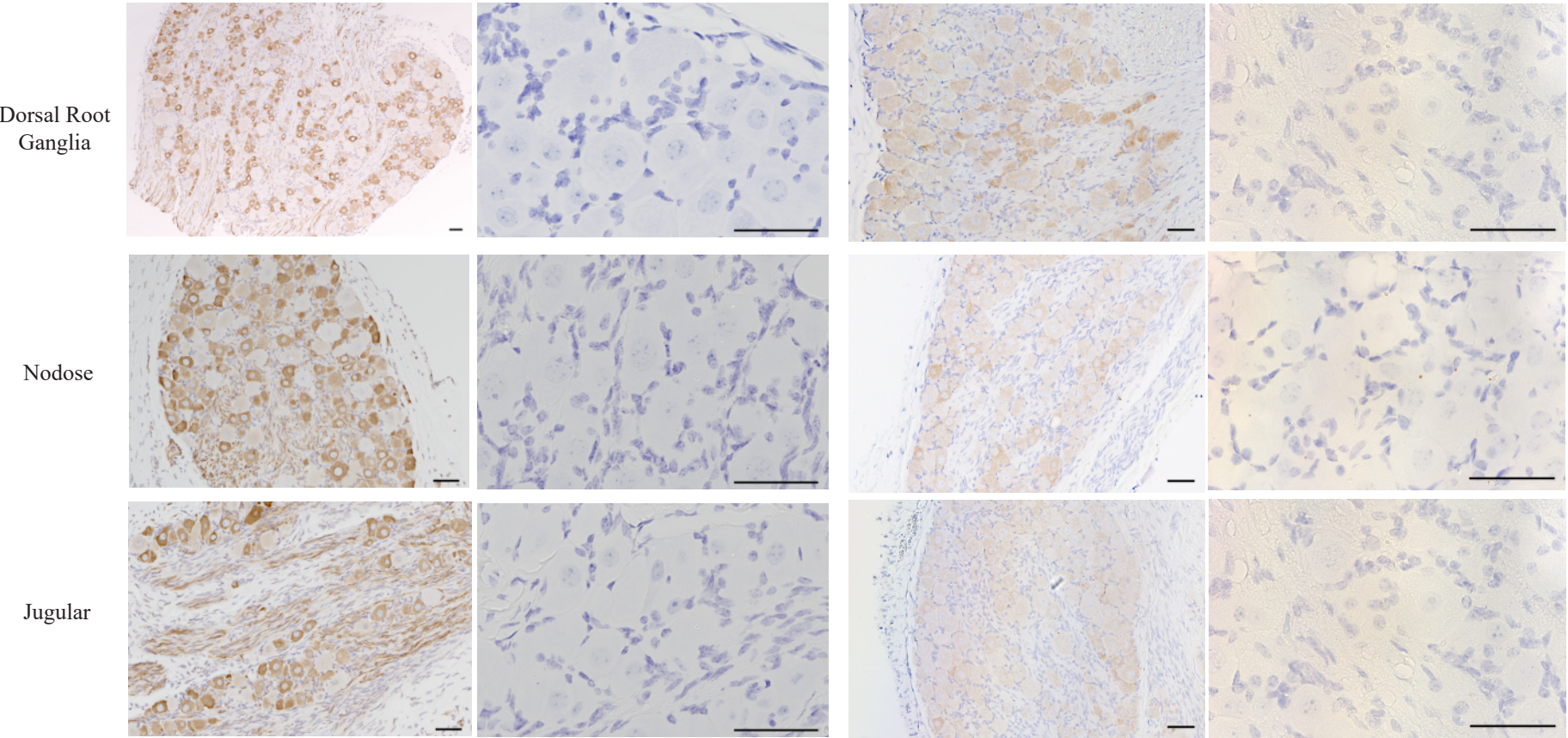

Supplement: Supplementary file 2 — Additional file 2: Figure S2. Immunohistochemistry with TLR7 antibodies indicates TLR-independent labeling of sensory neurons. Wild type and TLR7 knock out (KO) mouse dorsal root ganglia and vagal ganglia were paraffin embedded and processed for immunohistochemistry. Two separate TLR7 antibodies (TLR7 Ab-1, Novus; TLR7 Ab-2, Abcam) labeled small-to-medium-sized neurons (brown) in (A) dorsal root ganglia and (B) vagal ganglia in both wild type and TLR7 KO mice, and in (C) guinea pig dorsal root, nodose, and jugular ganglia. No primary antibody control experiments performed in parallel with TLR7 staining excluded non-specific secondary staining as the cause for neuronal labeling. Scale bar = 50 μm. Blue = hematoxylin nuclear stain. N = 4, representative images shown. [file 12974_2021_2269_MOESM2_ESM.pdf]
